# Afebrile brucellosis patients in endemic areas: a great diagnostic challenge

**DOI:** 10.3389/fmed.2026.1792416

**Published:** 2026-04-23

**Authors:** Yifan Ke, Shaoquan Zhang, Abuduweili Awuti, Maimaitiaili Tuerxun, Hong Cao, Yutian Chong

**Affiliations:** 1 Department of Infectious Diseases, The Third Affiliated Hospital of Sun Yat-sen University, Guangzhou, China; 2 Department of Infectious Diseases, The Affiliated Kashgar Hospital of Sun Yat-sen University, Kashi, China

**Keywords:** afebrile, *Brucella*, brucellosis, complication, infectious disease, zoonotic disease

## Abstract

**Background:**

Brucellosis is a common zoonotic infection in pastoral areas. The absence of fever in some patients can lead to misdiagnosis or underdiagnosis, resulting in delayed treatment. However, no literature have summarized the clinical features of afebrile brucellosis, and this study aimed to fill in the research gap.

**Methods:**

In this retrospective case–control study, patients diagnosed with brucellosis who were admitted to the Affiliated Kashgar Hospital of Sun Yat-sen University in 2024 were included. They were divided into afebrile and febrile groups. Demographic data, clinical features, and laboratory findings were compared between the two groups.

**Results:**

A total of 70 afebrile and 119 febrile patients were included in the study. Compared with febrile patients, afebrile patients had a higher proportion of chronic disease course (20% vs. 10%, *p* = 0.008) and were more likely to lack a clear epidemiological history (29% vs. 46%, *p* = 0.025). Afebrile patients were more likely to present with focal infections and localized symptoms, mainly spondylitis (51% vs. 24%, *p* < 0.001) and dorsalgia (66% vs. 42%, *p* = 0.003). In contrast, febrile patients were more likely to exhibit systemic toxic symptoms, mainly fatigue (75% vs. 34%, *p* < 0.001), inappetence (58% vs. 34%, *p* = 0.003), and hidrosis (37% vs. 17%, *p* = 0.007). The positive rate of bacterial culture was lower in afebrile patients (40% vs. 66%, *p* = 0.001). Febrile patients exhibited significantly lower hemoglobin levels, platelet counts, serum calcium and phosphorus levels, but higher procalcitonin levels, elevated transaminase levels, higher γ-glutamyl transferase levels, and a prolonged prothrombin time (all *p* < 0.05).

**Conclusion:**

The atypical clinical manifestations, lower positive rate of bacterial culture, and fewer abnormal laboratory findings pose a greater diagnostic challenge of afebrile brucellosis. Despite causing less systemic impact, afebrile brucellosis may be associated with a worse prognosis. Physicians in pastoral areas should emphasize etiological diagnosis for afebrile patients presenting with dorsalgia.

## 1 Introduction

Brucellosis is a zoonotic infection caused by *Brucella*, which can be transmitted through the respiratory tract, digestive tract, or via direct contact. The disease is primarily endemic in pastoral areas. From 2006 to 2019, more than 97 countries reported human brucellosis, with incidence rates between 0.002 and 293.103 per 100,000 population (1). In China, its incidence has shown a general upward trend, particularly in northwestern areas such as Xinjiang and Inner Mongolia (2). In 2023, the incidence rate in Xinjiang reached 36.08 per 100,000 population, making it a major public health concern (3, 4).

Approximately 2–4 weeks after infection, *Brucella* enters the bloodstream and can spread to various organs via the mononuclear phagocyte system (5). Complications involving the osteoarticular, genitourinary, respiratory, and hematological systems develop in approximately 54.8% of patients (6). The clinical manifestations of brucellosis include shivering, fever, hidrosis, fatigue, myalgia, and arthralgia. Fever is the most common symptom, serving as both the main reason for patients seeking medical attention and a key diagnostic indicator. However, 16–39% of brucellosis patients are afebrile (7–11). This absence of fever makes clinicians more susceptible to overlook brucellosis in differential diagnosis, often resulting in delayed treatment. To date, no studies have specifically assessed the impact of the presence or absence of fever on brucellosis patients.

Therefore, we performed a retrospective case–control study of brucellosis patients in Kashgar, Xinjiang. This study aimed to summarize the clinical features of afebrile patients, to improve clinicians’ recognition of this condition, thereby facilitating early diagnosis and treatment.

## 2 Materials and methods

### 2.1 Study population

Patients hospitalized with brucellosis at the Affiliated Kashgar Hospital of Sun Yat-sen University from January to December 2024 were included consecutively. Patients were divided into a case group (body temperature <37.3 °C before and throughout hospitalization) and a control group. The two groups were compared in terms of general characteristics, clinical manifestations, and laboratory findings.

### 2.2 Diagnostic criteria

#### 2.2.1 Inclusion criteria

(1) Residence in an endemic area, occupation in animal husbandry, or a history of contact with livestock or animal products; (2) Presence of clinical manifestations such as shivering, fever, hidrosis, fatigue, myalgia, or arthralgia; (3) Positive Rose Bengal plate agglutination test (RBPT) or enzyme-linked immunosorbent assay (ELISA) results; (4) Standard tube agglutination test (SAT) titer ≥1:100; (5) Isolation of *Brucella* from blood, bone marrow, pus, or other specimens. All included patients met criteria 1 and 2 plus at least one of criterion 3–5 above.

#### 2.2.2 Exclusion criteria

(1) A history of previous diagnosis and systematic treatment for brucellosis; (2) Comorbidity with other severe systemic diseases (e.g., malignancy); (3) Pregnancy or lactation.

#### 2.2.3 Complications

A focal infection confirmed by localized symptoms and results of imaging examinations or puncture biopsy.

### 2.3 Statistical analysis

Statistical analyses were performed using R software (Version 4.4.1). Continuous variables conforming to a normal distribution were expressed as mean ± standard deviation and compared using Student’s *t*-test. Other continuous variables were expressed as median (interquartile range) and compared using the Mann–Whitney *U* test. Categorical variables were presented as frequency (percentage) and compared using the chi-square test or Fisher’s exact test, as appropriate. A two-sided *p*-value < 0.05 was considered statistically significant. Missing values were imputed using the k-nearest neighbor (KNN) algorithm. The proportion of missing data was reported in Supplementary Table 1.

### 2.4 Ethics approval statement

This study was approved by the Ethics Committee of the Affiliated Kashgar Hospital of Sun Yat-sen University (Approval No.: (2025)34).

## 3 Results

A total of 189 brucellosis patients fulfilling the diagnostic criteria were admitted to the Affiliated Kashgar Hospital of Sun Yat-sen University from January to December 2024. They were divided into a case group (*n* = 70, 37%) and a control group (*n* = 119, 63%).

### 3.1 General characteristics

The general characteristics of the patients are summarized in Table 1. Most patients were male (n = 125, 66%), with a median age of 48 (32, 60) years. A significantly lower proportion of patients in the case group had a clear epidemiological history (either occupation in animal husbandry or contact with livestock or animal products) compared with the control group (29% vs. 46%, *p* = 0.025). The distribution of disease course differed significantly between the groups [acute (<90 days):53% vs. 75%; subacute (90–180 days): 27% vs. 15%; chronic (>180 days): 20% vs. 10%; *p* = 0.008]. Sex ratio, median age, body mass index, occupational distribution, and the proportion of comorbidities were comparable between the groups (*p* > 0.05).

**Table 1:** General characteristics.

Variables	Case group (*n* = 70)	Control group (*n* = 119)	*p*
Sex, *n* (%)			0.948
Male	47 (67)	78 (66)	
Female	23 (33)	41 (34)	
Age, years	50.5 (30.25, 60)	47 (33, 61)	0.989
BMI, kg/m^2^	23.38 ± 4.73	22.87 ± 4.63	0.471
Job, *n* (%)	farmer, 44 (63)	farmer, 68 (57)	0.536
Comorbidities, *n* (%)	25 (36)	44 (37)	0.986
Epidemiological history, *n* (%)	20 (29)	55 (46)	**0.025**
Course of disease, *n* (%)			0.008
Acute	37 (53)	89 (75)	
Subacute	19 (27)	18 (15)	
Chronic	14 (20)	12 (10)	

Comorbidities: Chronic diseases requiring long-term medication administration. Epidemiological history: Engagement in animal husbandry or close contact with livestock or animal products. Course of disease: Acute refers to within 3 months; Subacute refers to within 3–6 months; Chronic refers to more than 6 months. The bold values indicate statistically significant variables (*p* < 0.05).

### 3.2 Clinical manifestations

Clinical manifestations are presented in Table 2. Among all patients, the most common symptom was fever (63%), followed by fatigue (60%), dorsalgia (51%), inappetence (49%), arthralgia (34%), hidrosis (30%), respiratory symptoms (24%), headache (17%), marasmus (17%), digestive symptoms (8%), urinary symptoms (4%), and hemorrhagic symptoms (2%). The rate of dorsalgia was significantly higher in the case group than in the control group (66% vs. 42%, *p* = 0.003). In contrast, the rates of fatigue (34% vs. 75%, *p* < 0.001), hidrosis (17% vs. 37%, *p* = 0.007), inappetence (34% vs. 58%, *p* = 0.003), headache (7% vs. 23%, *p* = 0.011), and respiratory symptoms (10% vs. 33%, *p* < 0.001) were significantly lower in the case group than in the control group. The rates of other symptoms were comparable between the groups (*p* > 0.05).

**Table 2:** Clinical manifestations.

Variables	Case group (*n* = 70)	Control group (*n* = 119)	*p*
Fatigue, *n* (%)	24 (34)	89 (75)	**<0.001**
Arthralgia, *n* (%)	30 (43)	35 (29)	0.085
Dorsalgia, *n* (%)	46 (66)	50 (42)	**0.003**
Marasmus, *n* (%)	11 (16)	21 (18)	0.888
Hidrosis, *n* (%)	12 (17)	44 (37)	**0.007**
Inappetence, *n* (%)	24 (34)	69 (58)	**0.003**
Headache, *n* (%)	5 (7)	27 (23)	**0.011**
Hemorrhagic symptoms, *n* (%)	1 (1)	3 (3)	1.000
Digestive symptoms, *n* (%)	4 (6)	12 (10)	0.440
Respiratory symptoms, *n* (%)	7 (10)	39 (33)	**<0.001**
Urinary symptoms, *n* (%)	4 (6)	4 (3)	0.471

Marasmus: Body weight loss >3Kg in 1 month. Hemorrhagic symptoms: Petechiae, ecchymoses, gingival bleeding, epistaxis, etc. Digestive symptoms: Celialgia, ventosity, jaundice, etc. Respiratory symptoms: Cough, expectoration, dyspnea, chest tightness, etc. Urinary symptoms: Urinary irritation symptoms, dysuria, perineal pain, etc. The bold values indicate statistically significant variables (*p* < 0.05).

### 3.3 Complications

Complications are presented in Table 3. A total of 108 complications were observed (57%), including spondylitis (*n* = 64, 34%), arthritis (*n* = 35, 19%), epididymitis (*n* = 6, 3%), meningitis and pneumonia (2 cases each, 1%), and one case each of endocarditis, peritonitis, and dermatitis (0.5% each). Both the overall complications rate (77% vs. 45%, *p* < 0.001) and spondylitis rate (51% vs. 24%, *p* < 0.001) were significantly higher in the case group than in the control group. The rates of other complications were comparable between the two groups (*p* > 0.05).

**Table 3:** Complications.

Variables	Case group (*n* = 70)	Control group (*n* = 119)	*p*
Total, *n* (%)	54 (77)	54 (45)	**<0.001**
Spondylitis, *n* (%)	36 (51)	28 (24)	**<0.001**
Arthritis, *n* (%)	14 (20)	21 (18)	0.835
Epididymitis, *n* (%)	3 (4)	3 (3)	0.672
Meningitis, *n* (%)	0	2 (2)	0.531
Endocarditis, *n* (%)	1 (1)	0	0.370
Peritonitis, *n* (%)	1 (1)	0	0.370
Pneumonia, *n* (%)	0	2 (2)	0.531
Dermatitis, *n* (%)	0	1 (1)	1.000

The bold values indicate statistically significant variables (*p* < 0.05).

### 3.4 Laboratory findings

Laboratory findings are presented in Table 4. The positive rate of bacterial culture (including all specimens) was significantly lower in the case group than in the control group (40% vs. 66%, *p* = 0.001). Levels of procalcitonin (PCT), alanine aminotransferase (ALT), aspartate aminotransferase (AST), γ-glutamyl transferase (GGT), and prothrombin time (PT) were significantly higher in the control group than in the case group. Hemoglobin (HGB) levels, serum calcium levels, serum phosphorus levels, and platelet count (PLT) were significantly lower in the control group than in the case group. The remaining laboratory findings showed no significant intergroup differences (*p* > 0.05).

**Table 4:** Laboratory findings.

Variables	Case group (*n* = 70)	Control group (*n* = 119)	*p*
Culture			0.001
**Positive, *n* (%)**	**28 (40)**	**78 (66)**	
**Negative, *n* (%)**	**42 (60)**	**41 (34)**	
WBC (10^9^/L)			0.106
>9.5, *n* (%)	6 (9)	7 (6)	
<3.5, *n* (%)	3 (4)	16 (13)	
N%			0.994
>75, *n* (%)	5 (7)	9 (8)	
<40, *n* (%)	7 (10)	12 (10)	
L%			0.987
>50, *n* (%)	7 (10)	12 (10)	
<20, *n* (%)	6 (9)	11 (9)	
HGB (g/L)			0.003
**<120, *n* (%)**	**29 (41)**	**77 (65)**	
PLT (10^9^/L)			<0.001
**>350, *n* (%)**	**4 (6)**	**14 (12)**	
**<125, *n* (%)**	**2 (3)**	**24 (20)**	
PCT (ng/mL)			0.048
**≥0.046, *n* (%)**	**46 (66)**	**95 (80)**	
CRP (mg/L)			0.525
>4, *n* (%)	60 (86)	107 (90)	
ESR (mm/h)			0.257
>20, *n* (%)	56 (80)	85 (71)	
ALT (U/L)			0.005
**>40, *n* (%)**	**19 (27)**	**56 (47)**	
**<5, *n* (%)**	**1 (1)**	**0**	
AST (U/L)			0.002
**>40, *n* (%)**	**12 (17)**	**47 (39)**	
TBIL (μmol/L)			0.523
>19, *n* (%)	3 (4)	10 (8)	
<3.4, *n* (%)	4 (6)	5 (4)	
DBIL (μmol/L)			0.266
>6.8, *n* (%)	4 (6)	14 (12)	
GGT (U/L)			0.011
**>50, *n* (%)**	**21 (30)**	**60 (50)**	
**<11, *n* (%)**	**1 (1)**	**3 (3)**	
ALP (U/L)			0.742
>150, *n* (%)	14 (20)	29 (24)	
<40, *n* (%)	0	1 (1)	
ALB (g/L)			0.971
<40, *n* (%)	12 (17)	22 (18)	
GLB (g/L)			0.549
>40, *n* (%)	5 (7)	13 (11)	
Cre (μmol/L)			0.621
>115, *n* (%)	0	3 (3)	
<62, *n* (%)	40 (57)	66 (55)	
P (mmol/L)			0.028
**>1.55, *n* (%)**	**8 (11)**	**10 (8)**	
**<0.81, *n* (%)**	**0**	**10 (8)**	
Ca (mmol/L)			<0.001
**<2.08, *n* (%)**	**9 (13)**	**55 (46)**	
TG (mmol/L)			0.628
>1.7, *n* (%)	13 (19)	27 (23)	
TC (mmol/L)			0.940
>5.2, *n* (%)	3 (4)	7 (6)	
<3, *n* (%)	4 (6)	8 (7)	
LDLC (mmol/L)			0.185
>3.1, *n* (%)	14 (20)	14 (12)	
HDLC (mmol/L)			0.285
>2, *n* (%)	1 (1)	1 (1)	
<0.9, *n* (%)	42 (60)	84 (71)	
PT (s)			0.023
**>14, *n* (%)**	**2 (3)**	**17 (14)**	
INR			0.780
>1.5, *n* (%)	0	2 (2)	

WBC, white blood cells; N%, percentage of neutrophils; L%, percentage of lymphocytes; HGB, hemoglobin; PLT, platelet; PCT, procalcitonin; CRP, C-reaction protein; ESR, erythrocyte sedimentation rate; ALT, alanine aminotransferase; AST, aspartate aminotransferase; TBIL, total bilirubin; DBIL, direct bilirubin; GGT, gamma-glutamyl transpeptidase; ALP, alkaline phosphatase; ALB, serum albumin; GLB, serum globulin; Cre, serum creatinine; P, serum phosphorus; Ca, serum calcium; TG, serum triglyceride; TC, total cholesterol; LDLC, low density lipoprotein cholesterol; HDLC, high density lipoprotein cholesterol; PT, prothrombin time; INR, international standardized ratio. The bold values/terms indicate statistically significant variables (*p* < 0.05).

## 4 Discussion

In this study, the sex distribution (male: 66.1% vs. 66.6%) and mean age (45.4 years vs. 41.3 years) of brucellosis patients were comparable to those reported in a retrospective, cross-sectional study from our center from 2019 to 2023 (11). However, the proportion of farmers among patients was lower than that in the previous study (59.2% vs. 75.8%). Given that farmers are frequently exposed to livestock and thus at higher risk of infection, this decline may indicate effective prevention of brucellosis as both an occupational and a zoonotic disease (12). Health education should be strengthened to prevent infection among residents in endemic areas, particularly regarding the practice of consuming raw milk (13).

The proportion of patients with a clear epidemiological history was significantly lower in the case group than in the control group. Epidemiological history is a key diagnostic basis for brucellosis, and its absence in afebrile cases may mislead clinical diagnosis. Besides, afebrile patients are easier to forget their contact history with brucella, so this difference may due to recall bias. The significantly longer disease course in the case group suggests that such patients tend to delay seeking medical attention. A previous study indicated that a longer disease course in brucellosis is associated with a poorer prognosis (14).

Fever, fatigue, and inappetence are the most common systemic symptoms of brucellosis, whereas dorsalgia and arthralgia are the most common localized symptoms. The rates of fatigue, hidrosis, inappetence, headache, and respiratory symptoms were all significantly higher in the control group than in the case group. This indicates that febrile patients primarily present with systemic toxic symptoms. In contrast, afebrile patients often have an insidious onset and atypical symptoms, making clinical diagnosis more challenging. Importantly, the rate of dorsalgia was significantly higher in the case group than in the control group. As dorsalgia has been identified as an independent risk factor for a poor prognosis in brucellosis (14), clinicians in endemic areas who encounters patients with this symptom should consider brucellosis in the differential diagnosis and perform serological testing, regardless of fever status. Further evaluation with spinal CT or MRI is recommended to confirm the diagnosis and assess the severity of brucellar spondylitis (15, 16). In this study, 95 patients (50%) had underwent spinal CT or MRI, while 96 patients (51%) had suffered dorsalgia.

The overall complication rate in this study was higher than that in the previous study from our center (57% vs. 48%) (9). Reported complication rates in the literature vary widely, ranging from 26 to 90% (6, 10, 14, 17), with brucellar spondylitis rates ranging from 2 to 77% (18). The complication rate was significantly higher in the case group than in the control group, primarily due to the higher incidence of brucellar spondylitis. In endemic areas, brucellar spondylitis is a major cause of prolonged disease course and mortality in brucellosis (19). Furthermore, focal infection has been identified as an independent risk factor for treatment failure (17). Therefore, afebrile brucellosis patients are more likely to have a poor prognosis.

Among the laboratory findings, a high rate of abnormal values was observed for albumin (ALB), high-density lipoprotein cholesterol (HDL-C), creatinine (Cre), HGB, GGT, and ALT. Consistent with previous reports, brucellosis is associated with hypoalbuminemia (20), anemia (21), and decreased HDL-C levels (22). Hypoalbuminemia, in particular, is an independent risk factor for treatment failure (17). The observed low Cre levels was consistent with those in the previous study at our center (56% vs. 64%) (11), which may warrant consideration of dose adjustments of relevant medications. Elevated GGT and ALT levels suggest potential liver dysfunction associated with brucellosis. Given that rifampicin is commonly used in brucellosis treatment, clinicians should be vigilant for drug-induced liver injury, select antibiotics based on liver function tests, and promptly initiate hepatoprotective therapy when indicated (23).

The positive rate of bacterial culture was significantly lower in the case group than in the control group. This may be due to the longer disease course in these patients. Additionally, a reduced body temperature may be directly proportional to a lower positive rate of bacterial culture. The proportions of abnormal results for multiple laboratory findings were also significantly lower in the case group than in the control group. This suggests that afebrile patients are less likely to present with anemia, liver injury, and coagulopathy, likely due to focal infections. These findings further highlight the diagnostic challenge associated with afebrile brucellosis patients.

In summary, the clinical characteristics of afebrile brucellosis patients are as follows: (1) a longer disease course; (2) a predominance of focal infections with atypical manifestations; (3) increased diagnostic difficulty; and (4) a propensity for a poorer prognosis. Consequently, clinicians should be vigilant for brucellosis in afebrile patients from endemic areas. A clear epidemiological history, proactive serological testing, and appropriate spinal imaging are essential for timely diagnosis. Furthermore, enhancing health education on the transmission routes of brucellosis in pastoral areas remains imperative.

This retrospective case–control study aimed to improve recognition of brucellosis and facilitate the early diagnosis and treatment of afebrile patients. Its main limitations include a retrospective and single-center design, the relatively small sample size, possible selection bias, and the lack of long-term outcome assessment. Therefore, these findings need to be validated by large-scale prospective cohort studies.

## Data Availability

The raw data supporting the conclusions of this article will be made available by the authors, without undue reservation.
