# Supplementary material for: Afebrile brucellosis patients in endemic areas: a great diagnostic challenge
Source: Front Med (Lausanne). 2026 Apr 23;13:1792416. doi: 10.3389/fmed.2026.1792416 (PMC13149063; doi:10.3389/fmed.2026.1792416)
Supplement: Supplementary file 1 [file Table_1.pdf]

Supplemental Table 1. The proportion of missing data.

Variables	Missing data	Proportion
BMI, kg/m^2	2	1.06%
PCT (ng/mL)	26	13.76%
CRP (mg/L)	38	20.11%
ESR (mm/h)	48	25.40%
AST (U/L)	1	0.53%
TBIL (μmol/L)	4	2.12%
DBIL (μmol/L)	8	4.23%
GGT (U/L)	14	7.41%
ALP (U/L)	13	6.88%
ALB (g/L)	4	2.12%
GLB (g/L)	7	3.70%
Cre (μmol/L)	5	2.65%
P (mmol/L)	17	8.99%
Ca (mmol/L)	4	2.12%
TG (mmol/L)	72	38.10%
TC (mmol/L)	74	39.15%
LDLC (mmol/L)	72	38.10%
HDLC (mmol/L)	73	38.62%
PT (s)	9	4.76%

INR	9	4.76%

PCT: Procalcitonin; **CRP:** C-reaction protein; **ESR:** Erythrocyte sedimentation rate; **AST:** Aspartate aminotransferase; **TBIL:** Total bilirubin; **DBIL:** Direct bilirubin; **GGT:** Gamma-glutamyl transpeptidase; **ALP:** Alkaline phosphatase; **ALB:** Serum albumin; **GLB:** Serum globulin; **Cre:** Serum creatinine; **P:** Serum phosphorus; **Ca:** Serum calcium; **TG:** Serum triglyceride; **TC:** Total cholesterol; **LDLC:** Low density lipoprotein cholesterol; **HDLC:** High density lipoprotein cholesterol; **PT:** Prothrombin time; **INR:** International standardized ratio.
